# Expression of S1P metabolizing enzymes and receptors correlate with survival time and regulate cell migration in glioblastoma multiforme

**DOI:** 10.18632/oncotarget.7366

**Published:** 2016-02-13

**Authors:** Sandra Bien-Möller, Sandra Lange, Tobias Holm, Andreas Böhm, Heiko Paland, Johannes Küpper, Susann Herzog, Kerstin Weitmann, Christoph Havemann, Silke Vogelgesang, Sascha Marx, Wolfgang Hoffmann, Henry W.S. Schroeder, Bernhard H. Rauch

**Affiliations:** ^1^ Department of Pharmacology, University Medicine Greifswald, Greifswald, Germany; ^2^ Department of Neurosurgery, University Medicine Greifswald, Greifswald, Germany; ^3^ Institute of Pathology, Department of Neuropathology, University Medicine Greifswald, Greifswald, Germany; ^4^ Institute for Community Medicine, University Medicine Greifswald, Greifswald, Germany

**Keywords:** glioblastoma multiforme, sphingosine-1-phosphate, S1P receptor signaling

## Abstract

A signaling molecule which is involved in proliferation and migration of malignant cells is the lipid mediator sphingosine-1-phosphate (S1P). There are hints for a potential role of S1P signaling in malignant brain tumors such as glioblastoma multiforme (GBM) which is characterized by a poor prognosis. Therefore, a comprehensive expression analysis of S1P receptors (S1P_1_-S1P_5_) and S1P metabolizing enzymes in human GBM (*n* = 117) compared to healthy brain (*n* = 10) was performed to evaluate their role for patient's survival. Furthermore, influence of S1P receptor inhibition on proliferation and migration were studied in LN18 GBM cells. Compared to control brain, mRNA levels of S1P_1_, S1P_2_, S1P_3_ and S1P generating sphingosine kinase-1 were elevated in GBM. Kaplan-Meier analyses demonstrated an association between S1P_1_ and S1P_2_ with patient's survival times. *In vitro*, an inhibitory effect of the SphK inhibitor SKI-II on viability of LN18 cells was shown. S1P itself had no effect on viability but stimulated LN18 migration which was blocked by inhibition of S1P_1_ and S1P_2_. The participation of S1P_1_ and S1P_2_ in LN18 migration was further supported by siRNA-mediated silencing of these receptors. Immunoblots and inhibition experiments suggest an involvement of the PI3-kinase/AKT1 pathway in the chemotactic effect of S1P in LN18 cells.

In summary, our data argue for a role of S1P signaling in proliferation and migration of GBM cells. Individual components of the S1P pathway represent prognostic factors for patients with GBM. Perspectively, a selective modulation of S1P receptor subtypes could represent a therapeutic approach for GBM patients and requires further evaluation.

## INTRODUCTION

The glioblastoma multiforme (GBM) is the most common primary brain tumor in adults. Despite an adjuvant radiochemotherapy in addition to surgery, the GBM is characterized by rapid tumor recurrence resulting in a poor prognosis with a median survival time of only 12 to 15 months [1, 2]. To date, no groundbreaking improvements in the therapeutic management of GBM have been achieved. As underlying reasons for relapses of GBM the resistance of glioma stem-like cells against the current therapy as well as the migration and invasion of GBM cells into adjacent brain regions are discussed [3]. A signaling molecule which is involved in proliferation, migration and invasion of a broad range of healthy and malignant cells is the sphingosine-1-phosphate (S1P) [4]. S1P is formed intracellularly from sphingosine in a reaction catalyzed by the two isoenzymes sphingosine kinase 1 and 2 (SphK1/2) [5]. The S1P phosphatases 1 and 2 (SGPP1/2) dephosphorylate S1P back to sphingosine whereas the S1P lyase (SGPL) mediates the irreversible cleavage to hexadecenal and phosphoethanolamine [6, 7]. Neurons and astrocytes can constitutively export S1P which supports the hypothesis that cells of the nervous system can be an origin of extracellular S1P [8, 9]. Beside its intracellular actions, the autocrine and paracrine effects of S1P are mediated by a family of five G-protein coupled receptors (S1P_1_–S1P_5_) which display tissue specific expression patterns with overlapping functions but also with some opposite effects [4, 10]. They signal via G_i/o_ (S1P_1_, S1P_2_ and S1P_5_), G_q_ (S1P_2_ and S1P_3_), and G_12/13_ (S1P_2_, S1P_3_, and S1P_5_) proteins particularly to the Ras/ERK, PI3K/Akt, and Rho/Rock signaling pathways [10]. The specific signaling pathways utilized by S1P and the biological consequences depend on the cellular expression levels of the respective S1P receptor subtypes. Since S1P circulates in blood and lymphatic systems, it gains access to its receptors far away from the site of synthesis. Increased generation of S1P stimulates cell survival and malignant transformation, and regulates apoptosis, invasion, angiogenesis as well as hypoxia [4, 11–13]. Numerous studies have shown that SphK and S1P act as oncogenes in various tumors including lung, colon, breast, ovary, brain, stomach, uterus and kidney [13, 14]. Furthermore, an attenuation of tumor progression in murine xenograft and allograft models through administration of a specific S1P targeted monoclonal antibody was demonstrated [15].

In GBM the S1P receptors S1P_1_, S1P_2_, S1P_3_ and S1P_5_ are found to be overexpressed whereas S1PR_4_ is missing in GBM cells [16]. S1P levels also are strongly elevated in GBM tissue compared to non-malignant brain [17] and glioma cell lines are able to release S1P into the extracellular space [18]. The GBM stem cell survival and the regulation of a characteristic phenotypic stem cell profile also seems to involve S1P signaling [19–21] and the sphingosine analogue FTY720 (fingolimod) slowed growth of intracranial xenograft tumors in nude mice and augmented the therapeutic effect of temozolomide [22], the standard chemotherapeutic compound for treatment of patients with GBM. Thus, several studies argue for a potential role of S1P signaling in GBM growth and progress but the existing data concerning the impact of S1P in GBM cell proliferation and migration partly differ in their conclusions [23–27].

This study represents a comprehensive analysis of the expression of S1P receptors and enzymes involved in S1P metabolism in human GBM samples in comparison to healthy tissue specimens and evaluated their role for patient's survival. Furthermore, the effects of S1P receptor inhibition and siRNA mediated knockdown on viability and migration as well as underlying signaling pathways were analyzed in LN18 GBM cells.

## RESULTS

### Expression of S1P receptors and S1P metabolizing enzymes in GBM and impact on patient's survival

**Figure 1:**
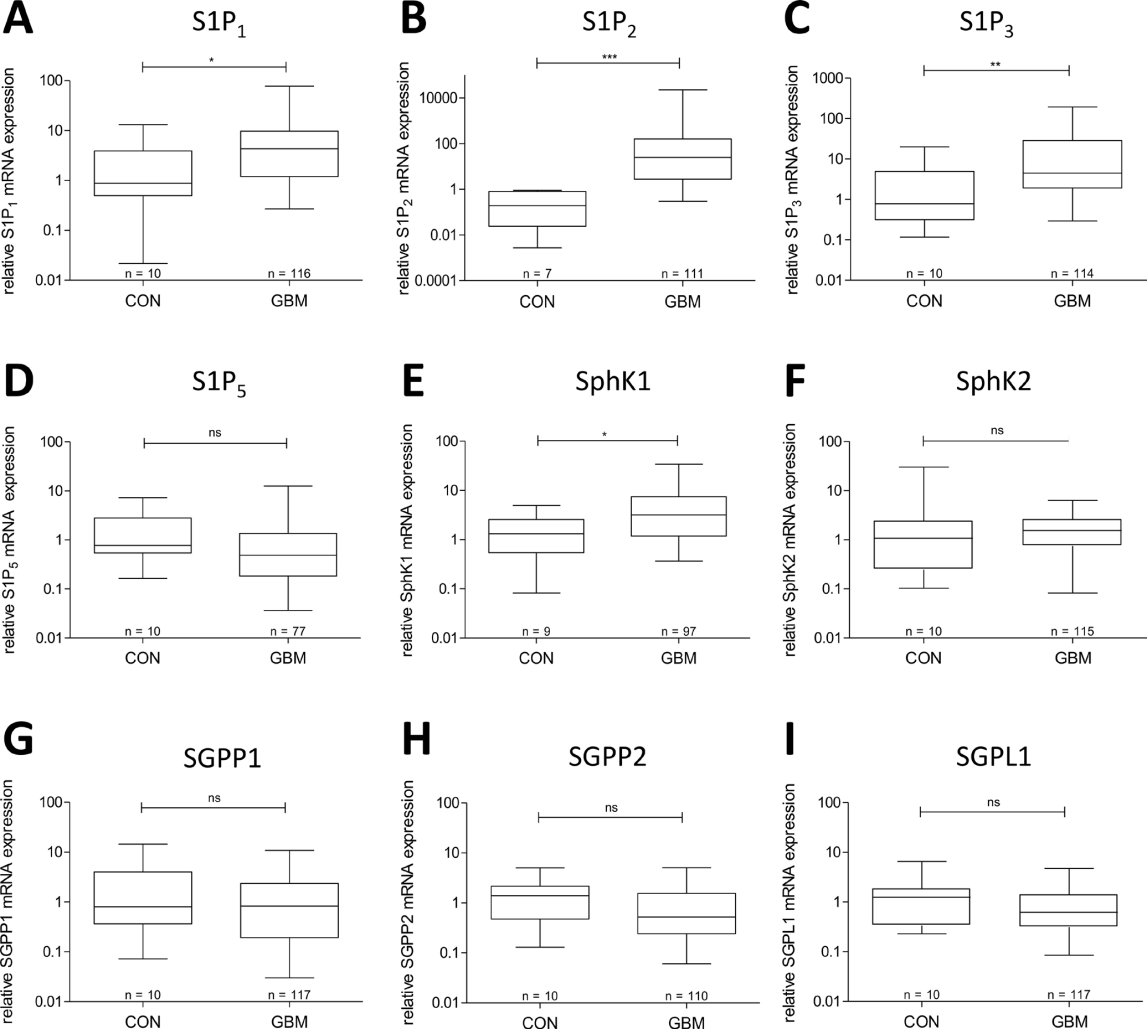
mRNA expression of S1P receptors and S1P metabolizing enzymes in glioblastoma tissue in comparison to non-malignant brain (**A**) S1P_1_ mRNA expression, (**B**) S1P_2_ mRNA expression, (**C**) S1P_3_ mRNA expression, (**D**) S1P_5_ mRNA expression, (**E**) SphK1 mRNA expression, (**F**) SphK2 mRNA expression, (**G**) SGPP1 mRNA expression, (**H**) SGPP2 mRNA expression, (**I**) SGPL1 mRNA expression. mRNA expression levels in frontal/temporal lobes of non-neoplastic brains (CON) and glioblastoma patient's samples (GBM) were analyzed by quantitative RT-PCR with normalization to 18S rRNA in relation to the median of CON. Data are shown as box plots representing the median as horizontal bars as well as the 5th and 95th percentile. Mann Whitney *U* test, ns = not significant, *p < 0.05, **p < 0.005 and ***p < 0.001.

Expression of S1P receptors and S1P metabolizing enzymes in GBM specimens was analyzed in comparison to non-malignant brain by quantitative RT-PCR. As seen in Figure 1, the mRNA expression levels of S1P_1_, S1P_2_ and S1P_3_ were significantly up-regulated compared to control brain from 0.88 to 4.23 (S1P_1_), from 0.19 to 24.78 (S1P_2_) and from 0.79 to 4.51 (S1P_3_) fold, respectively. In contrast, for S1P_5_ mRNA a slight down-regulation from 0.77 to 0.49 fold was observed in GBM specimens, and S1P_4_ expression was below the detection limit.

Concerning the S1P metabolizing enzymes, only the mRNA expression of the S1P generating enzyme SphK1 was significantly up-regulated in GBM compared to control brain from 1.31 to 3.18 fold. Expression of SphK2 as well as of the S1P degrading enzymes SGPP1/2 and SGPL1 in GBM was not significantly altered in comparison to that in non-malignant brain.

Interestingly, subdividing our patient cohort into primary tumors and relapses revealed no significant differences in the expression of S1P receptors or the S1P metabolizing enzymes (Supplementary Figure S1A–S1I).

**Figure 2:**
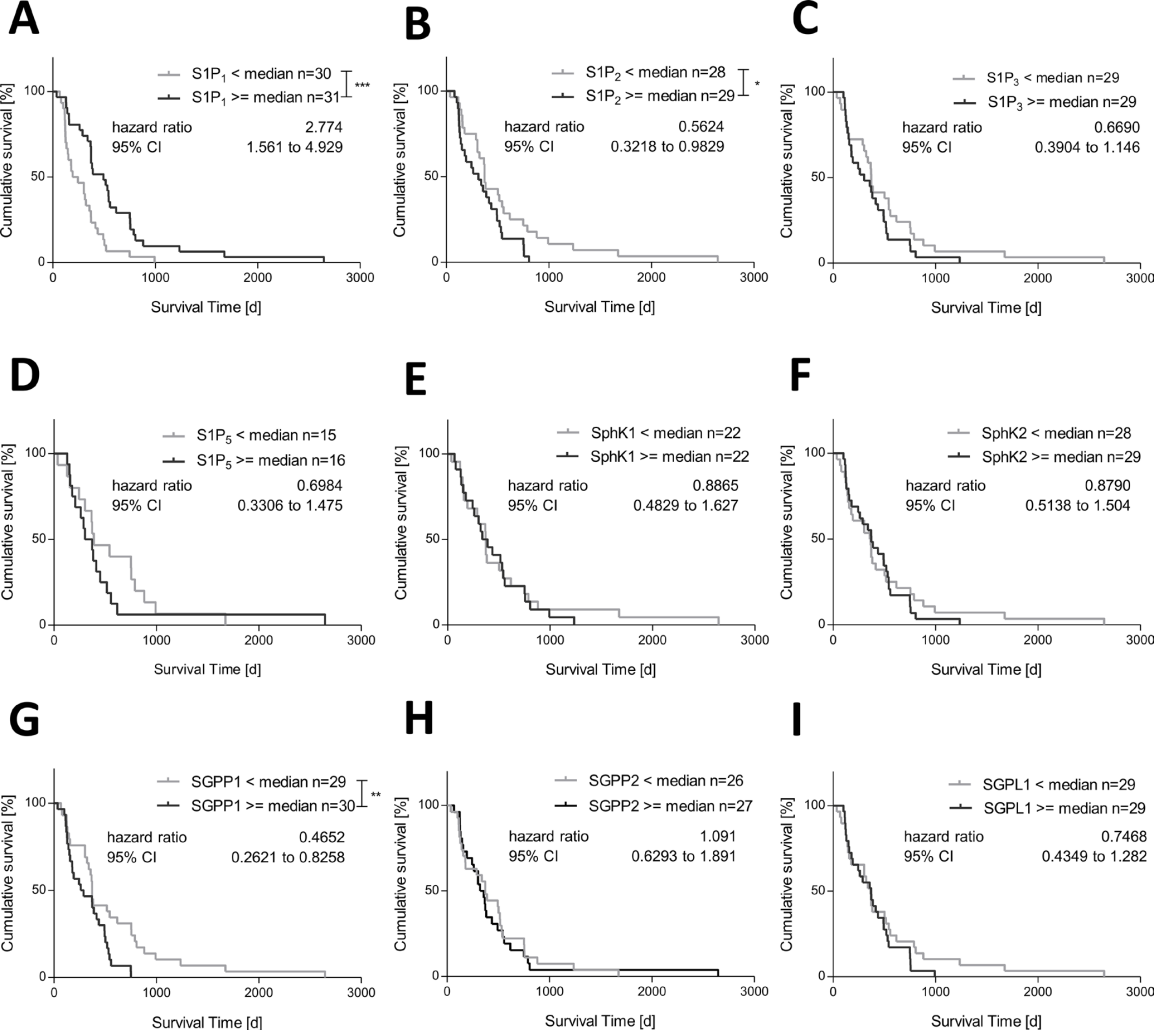
Association between mRNA expression of S1P receptors and S1P metabolizing enzymes and survival time of patients with glioblastoma multiforme Kaplan-Meier survival curves for patients with glioblastoma multiforme based on their (**A**) S1P_1_ mRNA expression, (**B**) S1P_2_ mRNA expression, (**C**) S1P_3_ mRNA expression, (**D**) S1P_5_ mRNA expression, (**E**) SphK1 mRNA expression, (**F**) SphK2 mRNA expression, (**G**) SGPP1 mRNA expression, (**H**) SGPP2 mRNA expression and (**I**) SGPL1 mRNA expression. Patients were divided into two subgroups depending on the respective median gene expression as determined by quantitative RT-PCR. Calculation of Hazard Ratios (< Median vs. > = Median expression), Log-rank (Mantel-Cox) test, *p < 0.05, **p < 0.005 and ***p < 0.001.

To investigate whether expression of S1P receptors and S1P metabolizing enzymes has any impact on the survival of patients with GBM we performed Kaplan-Meier analyses. Using the median gene expression the GBM patients were subdivided in two groups: < median versus > = median expression. As seen in Figure 2A and 2B, S1P_1_ and S1P_2_ expression was significantly associated with the survival time of GBM patients. Interestingly, for S1P_1_ a positive association with the patients' survival was observed. Patients with a high S1P_1_ mRNA expression (> = median) showed a prolonged survival compared to patients with a low S1P_1_ mRNA expression (< median, hazard ratio 2.77). In contrast, S1P_2_ mRNA expression negatively correlated with the survival time of GBM patients. Patients with a high S1P_2_ mRNA expression (> = median) had a worse survival in comparison to patients with a low S1P_2_ expression (< median, hazard ratio 0.56). Similarly, a high expression of S1P dephosphorylating SGPP1 (> = median) was associated with a poor survival compared to a low SGPP1 mRNA expression (< median, hazard ratio 0.47, Figure 2G). In contrast, expression of S1P_3_, S1P_5_, SphK1 and 2, SGPP2 or SGPL1 showed no association with the survival time of patients with GBM.

The observed prognostic relevance of S1P_1_ and S1P_2_ expression was maintained when the ratio between S1P_1_ and S1P_2_ expression (S1P_1_/S1P_2_) was used for survival analysis (hazard ratio 2.38, Supplementary Figure S2A). In addition, a moderate but significant negative correlation between S1P_1_ and S1P_2_ expression in GBM samples was seen (Spearman *r*−0.374, *p* = 0.0045, Supplementary Figure S2B). The subdivision of GBM patients in four subgroups according to high (> = median) and/or low (< median) S1P_1_ and S1P_2_ expression is shown in Supplementary Figure S2C–S2H. It was recognizable that the highest prognostic impact with a hazard ratio of 3.89 and a significantly prolonged survival was seen for the subgroup of GBM patients with a high expression of S1P_1_ combined with a low expression of S1P_2_ (Supplementary Figure S2H).

The combined survival analysis with the ratio between S1P_1_ and SGPP1 (S1P_1_/SGPP1) resulted in similar curves as seen for S1P_1_/S1P_2_, with a hazard ratio of 2.77 and a prolonged survival of patients with a high S1P_1_/SGPP1 ratio (< median, Supplementary Figure S3A). In contrast to S1P_1_ and S1P_2_, there was no correlation between the expression of S1P_1_ and SGPP1 (Supplementary Figure S3B). Interestingly, the highest hazard ratio of all survival analyses with a value of 5.76 and a significantly prolonged survival time was found for the subgroup with a high expression of S1P_1_ in combination with a low expression of SGPP1 (Supplementary Figure S3H) arguing for the use of the S1P_1_/SGPP1 ratio as best prognostic factor.

In addition, the simultaneous evaluation of S1P_2_ and SGPP1, which have a similar impact on survival of GBM patients (Figure 2B and 2G), with using the S1P_2_/SGPP1 ratio for survival analysis revealed no deviations in the compared curves (Supplementary Figure S4A). This is in line with the positive correlation between S1P_2_ and SGPP1 expression in GBM samples (Supplementary Figure S4B). Furthermore, a high hazard ratio with a value of 4.56 was seen for the subgroup with a high expression of S1P_2_ in combination with a low expression of SGPP1 (Supplementary Figure S4H).

**Figure 3:**
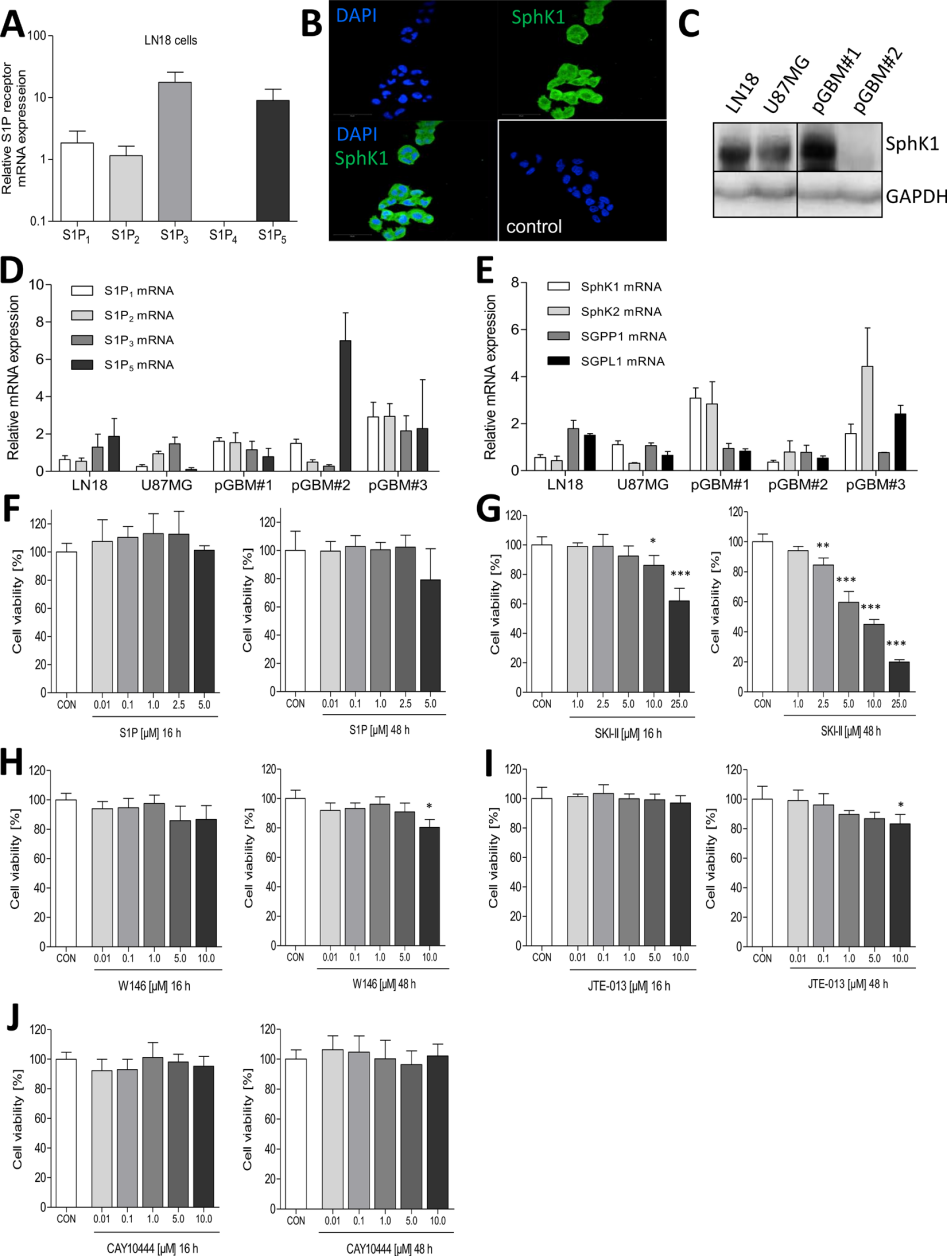
Analysis of the expression of S1P receptors as well as of S1P metabolizing enzymes and influence of its pharmacological blocking on cell viability of LN18 GBM cells (**A**) Relative mRNA expression of S1P receptors S1P_1_, S1P_2_, S1P_3_, S1P_4_ and S1P_5_ in the human LN18 GBM cell line determined by quantitative RT-PCR. (**B**) Immunofluorescence staining of the SphK1 in the human LN18 GBM cell line. The control (bottom right corner) represents LN18 cells stained only with the secondary AlexaFluor 488 coupled antibody without prior incubation with the primary anti-SphK1 antibody. (**C**) Immunoblot analysis of SphK1 protein expression in the human GBM cell lines LN18 and U87MG as well as in primary GBM cells (pGBM) isolated from fresh tumor samples. (D + E) Quantitative RT-PCR of (**D**) S1P receptors and (**E**) S1P metabolizing enzymes in the human GBM cell lines LN18 and U87MG as well as in primary GBM cells (pGBM) isolated from fresh tumor samples. (**F**–J). Determination of LN18 cell viability by using the resazurine assay after treatment with (C) S1P (0.01, 0.1, 1, 2.5 and 5 μM), (D) sphingosine kinase inhibitor SKI-II (1, 2.5,10 and 25 μM), (E) the S1P_1_ receptor antagonist W146 (0.01, 0.1, 1, 5 and 10 μM), (F) the S1P_2_ receptor antagonist JTE-013 (0.01, 0.1, 1, 5 and 10 μM) or (G) the S1P_3_ receptor antagonist CAY10444 (0.01, 0.1, 1, 5 and 10 μM) for 16 (left panel) or 48 h (right panel). Control cells (CON) were treated with the respective solvent (MeOH for S1P, DMSO for all inhibitors). Cell viability is shown in relation to the control (100%), mean values and SD, *n* = 3, One-way analysis of variance with Dunnett's multiple comparison test, *p < 0.05, **p < 0.005 and ***p < 0.001 vs. control.

Beside glioblastoma patient samples, we isolated glioblastoma cells from freshly resected tumor tissue of three different patients and analyzed the expression of S1P_1-5_, SphK1 and 2, SGPP1 and 2 as well as SGPL1 by quantitative RT-PCR. This was also performed in the human GBM cell lines LN18 and U87MG to compare and evaluate whether the tumor cells itself express all components of S1P signaling. As seen in Figure 3E, mRNA expression of SphK1/2, SGPP1 (but not SGPP2) and SGPL1 was detectable in the human cell lines LN18 and U87MG as well as in primary GBM cells (pGBM). Concerning S1P receptors, S1P_1-3_ and S1P_5_ are present in both GBM cell lines and primary cells (Figure 3D).

### Influence of S1P signaling on viability of LN18 GBM cells *in vitro*

For *in vitro* experiments we used the human GBM cell line LN18 which exhibits high mRNA expression levels of S1P_3_ and S1P_5_ and a lower expression of S1P_1_ and S1P_2_ whereas S1P_4_ was not found to be expressed (Figure 3A). This is consistent with the reported absence of S1P_4_ in GBM cells [16]. Expression of the S1P generating enzyme SphK1 in LN18 cells was determined by using immunofluorescence microscopy (Figure 3B). Furthermore, SphK1 protein expression was also strongly detectable by immunoblot analysis in the LN18 and U87MG cell lines as well as in one of the two investigated primary GBM cells (pGBM#1, Figure 3C).

Treatment of LN18 cells with different S1P concentrations (0.01 to 5 μM) for 16 h or 48 h did not result in any significant changes of cell viability (Figure 3F). In contrast, inhibition of SphK1/2 by SKI-II significantly reduced cell viability of LN18 cells. As shown in Figure 3G (left panel) 10 and 25 μM SKI-II significantly attenuated the viability of LN18 cells after 16 h to 86.1% and 61.9%, respectively. The effect of SKI-II on cell viability was greatly pronounced after 48 h by SKI-II concentrations of 5 μM, 10 μM or 25 μM SKI-II reducing cell viability to 59.6%, 45.0% and 19.9% (Figure 3G right panel). Even 2.5 μM SKI-II significantly reduced cell viability of LN18 cells to 84.5% compared with control cells. In comparison, inhibition of S1P_1_ or S1P_2_ by the compounds W146 or JTE-013 significantly reduced cell viability of LN18 cells to 80.4% and 83.3%, respectively, only after 48 h and at the highest concentration of 10 μM (Figure 3H and 3I). Incubation of LN18 cells with the S1P_3_ inhibitor CAY10444 at comparable concentrations did not affect the cell viability (Figure 3J).

### Influence of S1P signaling on migration of LN18 GBM cells

**Figure 4:**
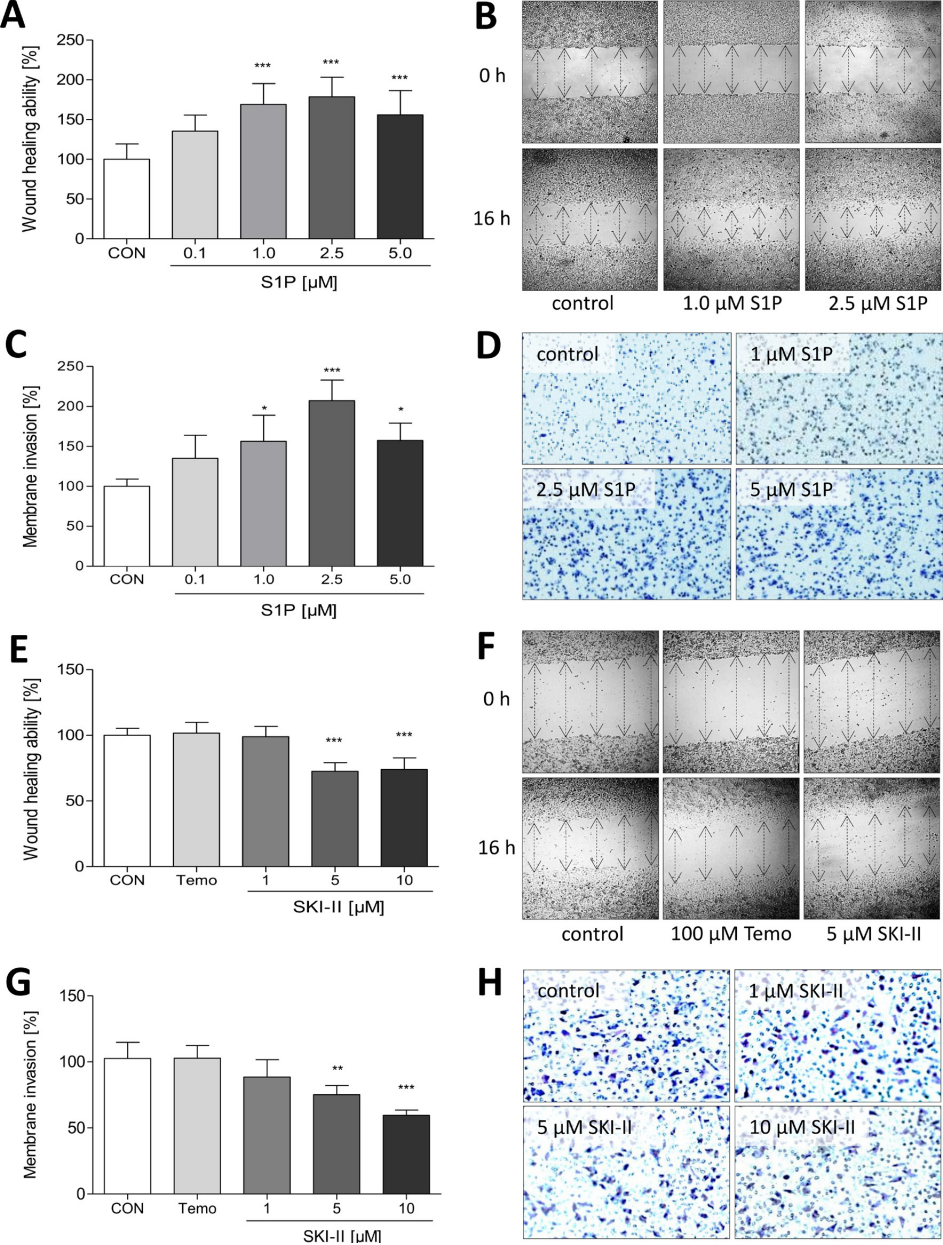
Influence of S1P, temozolomide and SKI-II on migration of LN18 GBM cells (**A** + **B**, **E** + **F**) Analysis of LN18 cell migration using the wound closure assay with setting a scratch into the cell layer and measurement of the wound width at the beginning of the experiment (0 h) and 16 h after treatment with S1P (A + B; 0.1, 1, 2.5 and 5 μM) and temozolomide (E + F; Temo, 100 μM) or SKI-II (E + F; 1, 5 and 10 μM). (A + E) Wound closure ability is shown in relation to the control (CON, 100%), mean values and SD, *n* = 3, One-way analysis of variance with Dunnett's multiple comparison test, ***p < 0.001 vs. control. (B + F) Representative images of the wound width at 0 h and 16 h, the wound width is marked by arrows. (C + D, **G** + **H**) Analysis of LN18 cell migration using the Boyden chamber assay. Cells were treated for 3 h with (C + D) S1P (0.1, 1, 2.5 and 5 μM) and (G + H) temozolomide (Temo, 100 μM) or SKI-II (1, 5 and 10 μM), fixed on the lower side of the membrane, stained with crystal violet and counted. (C + G) Counted cells are shown in relation to the control (CON, 100%), mean values and SD, *n* = 3, One-way analysis of variance with Dunnett's multiple comparison test, ***p < 0.001 vs. control. (D + H) Representative images of the migrated and stained cells after 3 h of stimulation.

Migration of LN18 cells was investigated with the scratch wound healing assay (16 h) and the Boyden chamber assay (3 h). As demonstrated in Figure 4A and 4B, incubation of LN18 cells with 1, 2.5 and 5 μM S1P significantly stimulated the migration of LN18 cells in the wound healing assay to 169%, 179% and 156%, respectively, compared to control cells. This pro-migratory effect of S1P was also seen in the Boyden chamber assay with a maximum effect of 207% at 2.5 μM S1P (Figure 4C and 4D). Conversely, inhibition of SphK by SKI-II (5 and 10 μM) resulted in a significantly reduced migration of LN18 cells to about 70% in the wound healing assay (Figure 4E and 4F). Again, this effect was confirmed in the Boyden chamber assay with migration values of 75% and 59.6% for 5 and 10 μM SKI-II, respectively (Figure 4G and 4H). In comparison, temozolomide as the standard chemotherapeutic agent for the treatment of GBM did not cause any alterations in the migration of LN18 cells.

**Figure 5:**
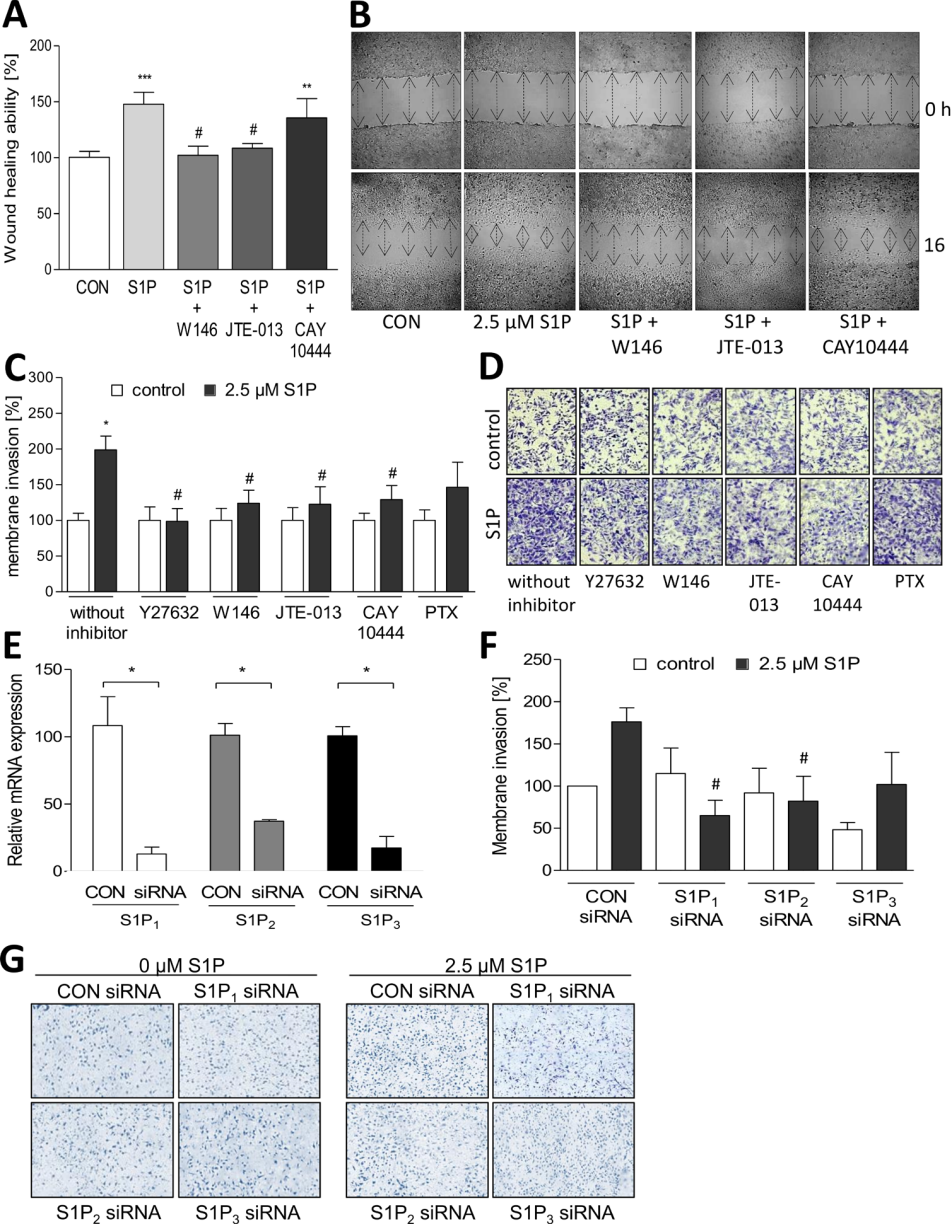
Association between S1P receptors and migration of LN18 GBM cells (**A** + **B**) Analysis of LN18 cell migration using the wound closure assay with setting a scratch into the cell layer and measurement of the wound width at the beginning of the experiment (0 h) and 16 h after treatment with MeOH (control), S1P alone (2.5 μM), S1P + 10 μM W146, S1P + 10 μM JTE-013 or S1P + 10 μM CAY10444. Cells were pre-treated for 1.5 h with W146, JTE-013, CAY10444 or DMSO (as solvent for the inhibitors) before adding S1P. (A) Wound closure ability is shown in relation to the control (100%), mean values and SD, *n* = 3, One-way analysis of variance with Dunnett's multiple comparison test, ***p < 0.001 vs. control, #*p* < 0.05 vs. S1P alone. (B) Representative images of the wound width at 0 and 16 h, the wound width is marked by arrows. (**C** + **D**) Analysis of LN18 cell migration using the Boyden chamber assay after inhibition of S1P_1_ with W146 (10 μM), S1P_2_ with JTE-013 (10 μM), S1P_3_ with CAY10444 (10 μM), p160ROCK with Y27632 (10 μM) or G_i/o_ signaling with Pertussis Toxin (PTX, 200 ng/ml). Cells were treated for 3 h with S1P (2.5 μM) with or without the indicated inhibitors, fixed on the lower side of the membrane, stained with crystal violet and counted. (C) Counted cells are shown in relation to the respective control (without inhibitor, 100%), mean values and SD, *n* = 3, *p < 0.05 vs. control, #*p* < *0.05* vs. S1P alone. (D) Representative images of the migrated and stained cells after 3 h of stimulation. (**E**) Relative gene expression of S1P_1_, S1P_2_ and S1P_3_ after siRNA mediated silencing of the respective S1P receptor subtype in LN18 GBM cells 48 h after transfection in comparison to control siRNA. mRNA expression levels were analyzed by quantitative RT-PCR with normalization to 18S rRNA and shown as mean values and SD, *n* = 3, Mann Whitney *U* test, *p < 0.05 vs. control siRNA (CON). (**F** + **G**) Analysis of LN18 cell migration using the Boyden chamber assay after siRNA mediated silencing of S1P_1_, S1P_2_ or S1P_3_ in comparison to control transfected cells. After 3 h of stimulation with S1P (2.5 μM), cells were fixed on the lower side of the membrane, stained with crystal violet and counted. (F) Counted cells are shown in relation to the control (100%), mean values and SD, *n* = 3, One-way analysis of variance with Dunnett's multiple comparison test, *p < 0.05 vs. control. (G) Representative images of the migrated and stained cells after 3 h of stimulation.

Further studies investigated the influence of S1P receptor inhibitors on GBM cell migration. As seen in Figure 5A and 5B, the S1P-induced increase in LN18 cell migration to 148% (2.5 μM S1P) was significantly reduced almost to control level by both inhibition of S1P_1_ with W146 and by blocking S1P_2_ with JTE-013 (10 μM, respectively) indicating that stimulation of both S1P_1_ and S1P_2_ is involved in the pro-migratory effect of S1P. In contrast, the S1P_3_ inhibitor CAY10444 did not significantly block S1P induced cell migration. Using the Boyden chamber assay, the inhibitory effect of W146 and JTE-013 was confirmed, but interestingly inhibition of S1P_3_ by CAY10444 also significantly diminished S1P induced migration of LN18 cells which differs from the results of the wound healing assay. Potentially, a role of S1P_3_ particularly for cell adhesion to the collagen coated membrane or the different time scale compared to the wound healing assay may account for this difference in the effects of S1P_3_ inhibition in the transmigration assay.

To analyze whether G_i/o_ protein or G_12/13_ and ROCK protein dependent action is responsible for the S1P receptor mediated effects, we treated LN18 cells with Pertussis Toxin (PTX) to inhibit G_i/o_ protein signaling or with the ROCK inhibitor Y27632. Treatment with Y27632 reverted the S1P induced LN18 cell migration to the control level whereas inhibition of G_i/o_ protein signaling by PTX reduced the pro-migratory effect of S1P to a much lesser and non-significant extent (Figure 5C and 5D).

To confirm the specific impact of the S1P receptor inhibitors on migration of GBM cells, we performed siRNA-mediated silencing of S1P_1_, S1P_2_ and S1P_3_ in LN18 cells. Compared to LN18 cells transfected with non-specific control siRNA, the mRNA levels of S1P_1_, S1P_2_ and S1P_3_ were significantly reduced by the respective receptor-specific siRNA constructs (Figure 5E) while the other receptor subtypes were not significantly influenced (Supplementary Figure S5). Using the Boyden chamber assay (Figure 5F and 5G), we found that the S1P-mediated increase in migration of LN18 cells was significantly reduced from 176% (2.5 μM S1P) to 65% and 85%, when S1P_1_ or S1P_2_ were silenced, respectively. In comparison, the siRNA-mediated knockdown of S1P_3_ did not cause a statistically significant decrease of cell migration.

### Analysis of the intracellular signaling pathway responsible for S1P-mediated cell migration

**Figure 6:**
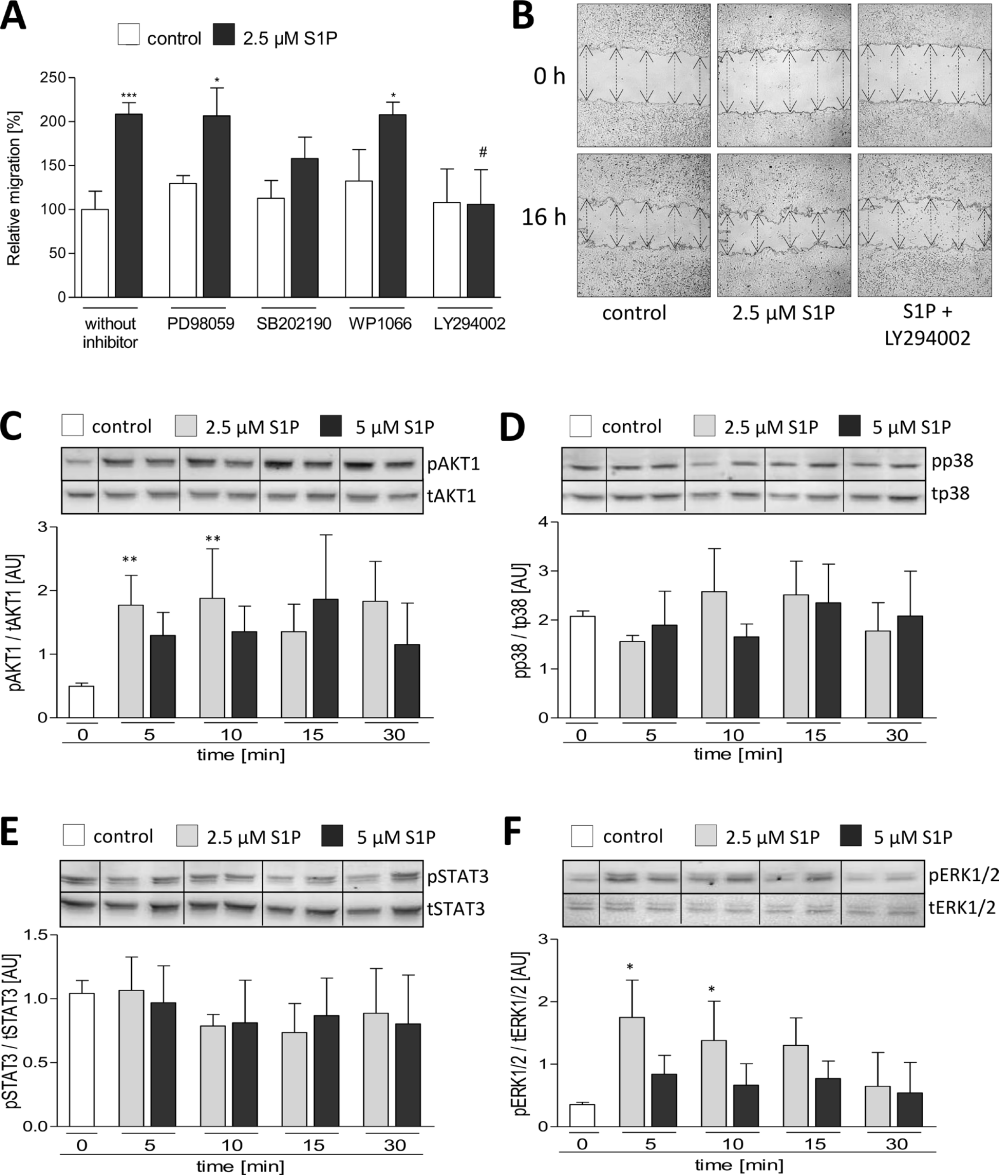
Association between signaling pathways and S1P induced migration of LN18 GBM cells (**A** + **B**) Analysis of LN18 cell migration using the wound closure assay with setting a scratch into the cell layer and measurement of the wound width at the beginning of the experiment (0 h) and 16 h after treatment with DMSO (solvent), 10 μM PD98059, 10 μM SB202190, 10 μM WP1066 or 10 μM LY294002 either in combination with MeOH (control) or 2.5 μM S1P. Cells were pre-treated for 1.5 h with PD98059, SB202190, WP1066, LY294002 or DMSO before adding S1P. (A) Wound closure ability is shown in relation to the control (100%), mean values and SD, *n* = 3, One-way analysis of variance with Dunnett's multiple comparison test, *p < 0.05 and ***p < 0.001 vs. control, #*p* < 0.05 vs. S1P alone. (B) Representative images of the wound width at 0 and 16 h, the wound width is marked by arrows. (C–F) Immunoblot analysis of the phosphorylated (p) and total (t) signaling molecules AKT1 (**C**), p38 (**D**), STAT3 (**E**) and ERK1/2 (**F**). LN18 GBM cells were treated with 2.5 or 5 μM S1P for 5, 10, 15 and 30 min. Representative blots of four independent experiments each. Densitometric analyses of four independent experiments, phosphorylated protein level (p) was normalized to the total protein level (t), mean values and SD. *p < 0.05 and **p < 0.005 vs. control.

To reveal which signaling pathways may be responsible for the S1P-mediated migration of LN18 GBM cells, we blocked known S1P-induced signaling pathways by specific inhibitors such as PD98059 (MEK/ERK inhibitor), SB202190 (p38 inhibitor), WP1066 (STAT3 inhibitor) and LY294002 (PI3K/AKT1 inhibitor). As seen in Figure 6A and 6B, only inhibition of the PI3K/AKT signaling by LY294002 almost completely abolished the S1P-mediated increase in LN18 cell migration from 208% (2.5 μM S1P alone) to 106%. The p38 inhibitor SB202190 also diminished the cell migration of LN18 cells to 158% but this effect was not statistically significant.

Immunoblot analyses showed a significant 4-fold increase in the phosphorylation status for both AKT1 (pAKT1) and ERK1/2 (pERK1/2) 5 and 10 min after stimulation of LN18 cells with 2.5 μM S1P (Figure 6C and 6F). In contrast, phosphorylation of p38 and STAT3 showed no alterations after treatment of LN18 cells with S1P (Figure 6D and 6E).

## DISCUSSION

Despite an aggressive multimodal therapy, the prognosis of patients with glioblastoma multiforme (GBM) is very poor with a 5-year survival rate of below 5% and a median patient life span of 12 to 15 month from diagnosis [1, 2]. The examination of signaling pathways which are responsible for the rapid recurrence are strongly necessary and might contribute to the optimization of the current therapeutic intervention.

The bioactive lipid S1P has been implicated in various disorders such as cancer and inflammatory diseases. Recent studies argue for a potential role of S1P signaling in GBM growth and progress but the available data concerning the impact of the S1P signaling system in GBM cell proliferation and migration partly differ in their conclusions [22–27].

The purpose of this study was to investigate the role of S1P receptors and S1P metabolizing enzymes for the prognosis of GBM patients as well as for proliferation and migration of GBM cells *in vitro*. Our data indicate that expression of S1P receptors S1P_1_, S1P_2_ and S1P_3_ as well as of SphK1 is up-regulated in GBM tissue in comparison to non-malignant brain whereas SphK2 was unchanged and a trend to a lower expression of S1P_5_ was observed in GBM. This is only partly in accordance with a recently published study which shows overexpression of SphK1/2, S1P_1_, S1P_2_ S1P_3_ and also S1P_5_ in GBM [16]. Conversely to the results of Quint and colleagues [16], another rather small study describes a down-regulation of S1P_1_ expression in GBM which correlates with a shorter survival of the patients [25]. These observations argue for certain variability in the expression of genes related to S1P signaling in heterogeneous tumors such as GBM and also indicates the need for larger patient cohorts. Another recent study from Abuhusain et al. demonstrated that an altered S1P/ceramide balance with reduced C18 ceramide and elevated S1P as well as increased SphK1 and decreased SGPP2 expression may be an important feature of human gliomas [17]. SGPP1 and SGPL were not examined in the study of Abuhusain and colleagues, therefore we additionally investigated the expression of these S1P degrading enzymes, however we found no significant alterations in GBM tissue and only a tendency of a reduced SGPP2 expression (*p* = 0.1917). To evaluate the role of the key players in S1P signaling in the prognosis of patients with GBM, Kaplan-Meier survival curves were calculated for patients divided in two subgroups depending on the median gene expression. These survival analyses showed a significant association between survival time and expression of S1P_1_, S1P_2_ and SGPP1 but not of S1P_5_ for which an prognostic impact was described by Quint and colleagues [16]. In agreement with the study from Yoshida et al. [25], a high expression of S1P_1_ also correlated with a prolonged survival of GBM patients in our study. This was unexpected since S1P is known to stimulate tumor cell proliferation and migration through binding to S1P_1_. Nevertheless, siRNA-mediated silencing of S1P_1_ in T98G and G112 glioma cells resulted in promoted cell proliferation [25]. Furthermore forced expression of S1P_1_ actually led to a reduced tumor growth in transplanted gliomas *in vivo* [25]. Thus, it seems possible that, depending on the individual expression of S1P_1_ and the other S1P receptor subtypes and in combination with differentially activated intracellular signaling pathways, S1P can mediate both pro- and anti-tumorigenic effects. Our results do not argue for an extraordinary role of S1P_1_ in proliferation of LN18 glioma cells since the S1P_1_ inhibitor W146 only slightly decreased cell viability whereas potent function of S1P_1_ in S1P-mediated tumor cell migration was observed. The same effect could be shown for inhibition of S1P_2_ by JTE-013. In agreement with the pro-migratory effect of S1P_2_ in our *in vitro* experiments, a high expression level of S1P_2_ correlated with a poor survival outcome. Thus, it is possible that signaling effects of S1P_2_ *in vivo* are more important for cell migration and tumor invasion than the S1P_1_ function.

Furthermore, our survival analyses showed that low expression of the phosphatase SGPP1 is associated with a prolonged survival rate of patients with GBM. SGPP1 dephosphorylates S1P back to sphingosine and thereby reduces the local S1P levels and its mediated effects [28]. Beside its role in proliferation, S1P regulates diverse cellular processes that are important for immune responses including differentiation, trafficking and migration of numerous types of immune cells such as T and B lymphocytes, natural killer cells, macrophages, or haematopoietic progenitors as well as cytokine and chemokine production [29]. The GBM tissue involves not only the tumor cells itself but rather represents a complex formation of extracellular matrix and diverse non-malignant cells such as endothelial cells, microglia or immunocompetent cells from peripheral blood [30]. So it might be possible that the locally produced S1P attracts immune cells into the tumor region to attack the malignant cells being enhanced by a low expression of SGPP1 and thus higher S1P levels.

Glioma cell lines are able to produce and release S1P, express S1P receptors and also respond to this signaling molecule [18, 19, 23]. The LN18 GBM cells used in our study express S1P_1_, S1P_2_, S1P_3_ and S1P_5_ as well as SphK and therefore can be assumed as a suitable model for the investigation of S1P-mediated effects in GBM cells. Stimulation of cultured GBM cells with S1P either results in unchanged or enhanced cell proliferation [23, 24] mediated by S1P_1_, S1P_2_ and S1P_3_ whereas S1P_5_ inhibits S1P-stimulated cell proliferation [24]. In our study, S1P did not directly stimulate LN18 cell viability. Since LN18 cells express high levels of S1P_5_, for which a role in survival of GBM patients has been suggested [16], and to a minor extent expresses S1P_1_ and S1P_2_, proliferative effects of S1P via S1P_1-3_ could be counteracted by S1P_5_. As described above, blockade of S1P_1_ by W146 and S1P_2_ by JTE-013 only marginally impaired the viability of LN18 cells at a concentration of 10 μM whereas the S1P_3_ inhibitor CAY10444 had no influence. In contrast, inhibition of SphK with SKI-II significantly impaired viability of LN18 cells. This is in agreement with the recently described suppression of GBM growth by inhibition of SphK1 in an animal model [31]. In addition, cytotoxic effects of SphK inhibitors were also seen in Temozolomide-resistant glioma cells *in vitro* [32]. A recent study by Van Brocklyn et al. demonstrated in Kaplan-Meier survival analysis that expression of SphK1 is associated with the outcome of GBM patients [33] but such a relation between patient's survival and SphK1 expression was not detectable in our patient cohort despite an elevated expression of SphK1 in the investigated GBM specimens. The observed up-regulation of SphK1 is, however, in accordance with data from Yoshida and colleagues and this group also found no significant correlation of SphK expression with the histological grade or with patient survival.

Inhibition of SphK (by SKI-II), S1P_1_ (by W146), S1P_2_ (by JTE-013) but not S1P_3_ (by CAY10444) resulted in an enhanced migration of LN18 cells in our *in vitro* studies. Therefore one would expect that high expression of S1P_1_ and S1P_2_ in GBM specimens is associated with a poor prognosis. But as mentioned above this was only the case for S1P_2_ but not for S1P_1_. It has been shown before that motility of GBM cells is stimulated by S1P [26] and involves both S1P_1_ and S1P_3_. In contrast, S1P_2_ has been suggested to inhibit migration and motility [23, 27]. Interestingly, Young and Van Brocklyn demonstrated an opposite effect for the S1P_2_ receptor, actually enhancing invasion of GBM cells as also seen for S1P_1_ and S1P_3_ [24]. Our *in vitro* data using LN18 cells argue for a role of S1P_1_ and S1P_2_ but not of S1P_3_ in migration of GBM cells. W146 is described as a specific S1P_1_ antagonist at the concentrations used in our study [34, 35]. In contrast, the S1P_2_ inhibitor JTE-013 was shown to additionally antagonize S1P_4_, which was not expressed in GBM cells in our study, however additional off-target effects of JTE-013 cannot be excluded [36]. Furthermore, for the S1P_3_ inhibitor CAY10444 blocking of purinergic P2 receptor and α_A1_ adrenoceptor was shown [36]. Therefore, the results obtained with the S1P receptor inhibitors were validated by siRNA mediated down-regulation of S1P_1_, S1P_2_ and S1P_3_. Isolated knockdown of all three S1P receptors resulted in a reduced migration of LN18 GBM cells whereby suppression of S1P_1_ had the strongest effect, which is in accordance with the pharmacological inhibition by W146, followed by S1P_2_ silencing. In contrast to the pharmacological inhibition of S1P_3_ using CAY10444, siRNA-mediated silencing of this receptor subtype also resulted in a trend to a reduced migration of LN18 cells.

To further analyze which pathway is involved in S1P-stimulated migration of LN18 cells, various inhibitors of signaling pathways, known to be activated by S1P, were utilized. Only inhibition of PI3-kinase/AKT1 signaling by LY294002 completely inhibited S1P stimulated LN18 cell migration. This was confirmed by an increased phosphorylation of AKT1 after stimulation of the cells with S1P. The PI3-kinase/AKT1 pathway is known to be activated by S1P_1_, S1P_2_ and S1P_3_ via G_i_-coupling of these receptors [27]. For S1P_1_ and S1P_3_ a PI3-kinase/AKT1-dependent stimulation of cell migration is described whereas S1P_3_ uses this signaling cascade only as a side trail while Rho signaling as the main pathway may negatively regulate cell migration [27]. Nevertheless in our experiments, inhibition or specific silencing of S1P_2_ resulted in a significant reduction of migration of LN18 GBM cells. In accordance with this, glioma cell invasion was also increased by stimulation of S1P_2_ in a study by Young and Van Brocklyn [24].

In contrast to AKT1, STAT3 phosphorylation was not significantly changed after stimulation of LN18 cells with S1P despite a previously reported down-regulation of phosphorylated STAT3 in brain tumor stem cells after stimulation with the S1P analogue fingolimod [22]. For ERK1/2 we also found an increased phosphorylation after stimulation of LN18 cells with S1P but inhibition of this signaling cascade with PD98059 failed to reduce cell migration. Activation of ERK1/2 by S1P is known to increase growth and survival of GBM cells [13, 23] which was not observed in our GBM cell model. For inhibition of p38 kinase using SB202190, a tendency of a reduced LN18 cell migration was seen but the phosphorylation of p38 kinase was not increased after stimulation of the cells with S1P. Overall, our *in vitro* data argue for a significant role of the PI3-kinase/AKT1 pathway in S1P-mediated stimulation of GBM cell migration. This is in agreement with a recently published study showing that the sphingosine analogue FTY720 (fingolimod) reduces migration and invasion of GBM cells by blocking the PI3-kinase/AKT signaling axis [37].

In summary, our data argue for a participation of S1P signaling in proliferation and migration of GBM cells. Individual components of the S1P pathway represent prognostic factors for survival of patients with GBM. Additionally, our results implicate a very complex interplay between S1P receptors, S1P signaling and other tumor-promoting signaling cascades in GBM making it difficult to directly link *in vivo* to *in vitro* data. Perspectively, the selective inhibition of S1P receptor subtypes could represent a therapeutic approach for GBM patients and requires further evaluation.

## MATERIALS AND METHODS

### Patients samples

Following an institutional review board-approved protocol, fresh human GBM tissues were collected from 117 patients (70 males, 47 females) who underwent surgical removal of GBM within their therapeutic regime (study period from 15.10.2007 to 31.07.2014). The histological analyses are based on the World Health Organization criteria [38]. Tumor specimens were obtained from primary tumors (*n* = 77) and relapses (*n* = 40). The last update of the vital status was on July 31, 2014, delivering 67 full cases of deceased patients with primary tumor for subsequent survival analysis. Overall survival was measured from the date of diagnosis to the date of death. Table 1 shows the detailed clinical characteristics. Beside GBM samples, eight non-neoplastic brain tissues (frontal/temporal lobes) from the Institute of Pathology/Department of Neuropathology of the University Greifswald were analyzed. Brain tissues of these control cases were obtained by routine autopsy. After the brain was removed tissue samples were cut and frozen at minus 80°C immediately. The autopsy cases died of pneumonia, heart failure, sepsis or carcinoma of pancreas, respectively. There were no neurological disorders. Further, RNA samples of two non-malignant (one frontal and one temporal lobe) specimens were obtained from BioChain Institute Inc. (Newark, CA, USA).

**Table 1:** Clinico-pathological characteristics of tumor specimens

Characteristic	value
N (primary tumor; relapse)	117 (77; 40)
median age at diagnose (25th-percentile; 75th-percentile)	64 (54; 71)
age classes	
< 50 years	15 (12.8%)
50 - < 60 years	35 (29.9%)
60 - < 70 years	29 (24.8%)
70 - < 80 years	29 (24.8%)
> 80 years	9 (7.7%)
Gender	
male	70 (60.9%)
female	47 (40.9%)
male-to-female ratio	1.5
vital status at study end	
dead	67 (58.3%)
alive	50 (43.5%)
resection grade	
total	57 (49.6%)
subtotal	60 (52.2%)
therapy regimen^a^	
radiotherapy and chemotherapy	48 (41.0%)
only radiotherapy	15 (12.8%)
only temozolomide	8 (6.8%)
other regimen	11 (9.4%)
no adjuvant therapy	10 (8.5%)

a = 25 therapy regimens could not be accessed.

### Cell culture

For *in vitro* experiments we used the LN18 GBM cell line which was obtained from the American Type Culture Collection (ATCC, Manassas, VA, USA). LN18 cells were maintained in DMEM supplemented with 10% FCS, 2 mM glutamine and 2 mM non-essential amino acids at 37°C, 95% humidity and 5% CO_2_. All cell culture materials were from PAA Laboratories (Cölbe, Germany). For stimulation experiments with S1P and inhibitors, LN18 cells were cultured in DMEM containing 0.05% FCS since. Reagents for cell culture experiments were as follows: S1P, JTE-013, W146, (all Sigma-Aldrich, Deisenhofen, Germany), LY294002, PD98059, SB202190, WP1066, Y27632 (all Calbiochem, Bad Soden, Germany), CAY10444 (CAYMAN Chemicals, Michigan, USA) and Pertussis Toxin (Tocris Biosciences, Bristol, UK).

### Isolation of tumor cells from glioblastoma samples

Isolation of single-cell suspension from human glioblastoma samples was performed using the *Brain Tumor Dissociation Kit* (Miltenyi Biotech, Bergisch Gladbach, Germany) according to the manufacturer's protocol. Afterwards, the isolated tumor cells were cultured as adherent cells in DMEM supplemented with 10% FCS, 2 mM glutamine and 2 mM non-essential amino acids at 37°C, 95% humidity and 5% CO_2_. After the second passage, RNA or protein was isolated and expression of S1P_1-5_, SphK1 and 2, SGPP1 and 2 and SGPL1 was analyzed by quantitative RT-PCR or Western Blot in comparison to the human glioblastoma cell lines LN18 and U87MG.

### Quantitative real-time PCR analysis

For mRNA expression analysis, total RNA was isolated using PeqGold RNAPure (PeqLab, Erlangen, Germany) and reversely transcribed using the High Capacity cDNA Reverse Transcription Kit (Applied Biosystems by Life Technologies, Weiterstadt, Germany). The expression was analyzed by the following Gene Expression Assays on Demand from Applied Biosystems: S1P_1_, Hs00173499_m1; S1P_2_, Hs01015603_s1; S1P_3_, Hs01019574_m1; S1P_4_, Hs02330084_s1; S1P_5_, Hs009 24881_m1; SphK1, Hs00184211_m1; SphK2, Hs002199 99_m1; SGPP1, Hs00229266_m1; SGPP2, Hs0054 4786_ m1; SGPL1, Hs00393705_m1, and eukaryotic 18S rRNA endogenous control, 4310893E. Quantitative real-time PCR was performed in a 7900 HT Fast Real-Time PCR system from Applied Biosystems. Each mRNA level was normalized to 18S rRNA and analyzed by the ΔΔct method.

### Immunofluorescence microscopy

LN18 cells were seeded onto cover slips at a density of 50 000 cells/well in a 12-well multiplate in 1 ml culture medium for 24 h. After aspiration of the medium and three washing steps with PBS, cells were fixed in 4% paraformaldehyde for 10 min, rinsed with PBS three times and then permeabilized with 0.1% Triton X-100 for 10 min. Afterwards, cells were blocked in 5% FCS for 1 h, and the primary antibody against SphK1 (Abgent, Hamburg, Germany) was incubated at 4°C overnight in blocking solution at a dilution of 1:50. Following three washing steps with PBS for each 5 minutes, cells were incubated for 2 hours with the secondary Alexa Fluor 488 labeled goat anti-mouse antibody (life technologies, Darmstadt, Germany) at a dilution of 1:200. After three further washing steps with PBS for 5 minutes, cells were incubated for 10 min with DAPI (4′,6′-diamidino-2-phenylindole, diluted 1:1000 in PBS, life technologies, Darmstadt, Germany) for counterstaining of nuclei followed by washing with PBS for three times (5 min each). Finally, cells were embedded in Dako Fluorescence Mounting Medium (Dako North West Inc., Carpintera, USA) on a slide and dried flat overnight before being examined at the Zeiss LSM780 fluorescence microscope (Carl Zeiss Microscopy, Jena, Germany).

### Western blot analysis

Protein lysates from cultured cells were prepared by using the following lysis buffer: 50 mM Tris-HCl pH 7.4, 100 mM NaCl, 0.1% Triton X-100, 5 mM EDTA containing protease/phosphatase inhibitors (1 mM phenylmethylsulfonyl fluoride, 1 mM leupeptin, 1 mM aprotinin, and 250 μg/mL sodium vanadate). Briefly, after centrifugation of cells at 6000 rpm the resulting pellet was dissolved in the lysis buffer and incubated on ice for 45 min. The BCA Protein Assay Kit (Thermo Fisher Scientific) was used for the protein quantification. Subsequently, after denaturation in Laemmli buffer at 95°C for 5 min, 25 μg of each sample was separated on a 10% sodium dodecyl sulfate polyacrylamide gel. Whatman nitrocellulose membranes (Proton, Schleicher and Schuell, Dassel, Germany) were used for immunoblotting in a tank blot system (Biometra, Göttingen, Germany). The membrane was blocked in 5% skim milk in Tris-buffered saline containing 0.05% Tween 20 (TBST) for 1 h at room temperature. The following primary antibodies were diluted in TBST and 0.05% sodium azide and incubated either for 2 h at room temperature or overnight at 4°C: monoclonal rabbit anti-phosphorylated Akt1 (Ser473), monoclonal rabbit anti-Akt1, monoclonal rabbit anti-phosphorylated ERK1/2 (T201/Y204), monoclonal mouse anti-ERK1/2, monoclonal rabbit anti-phosphorylated p38 (T180/Y182), rabbit anti-p38, monoclonal mouse anti-phosphorylated STAT3 (Y705), monoclonal mouse anti-STAT3 (all from Cell Signaling Technology, Boston, USA). The secondary horseradish peroxidase conjugated goat anti-rabbit or goat anti-mouse IgG antibodies (Bio-Rad, Munich, Germany) were used at a 1:2000 dilution for 1.5 h at room temperature. The detection of chemiluminescence signals was carried out with the ChemiDoc XRS Imaging System (Bio-Rad, Munich, Germany) using ECL Plus Western Blotting Substrate (Amersham Biosciences, Freiburg, Germany) followed by densitometric analysis (Quantity One, Bio-Rad). The relative optical densities of the specific phosphorylated bands were calculated and normalized to the respective total kinase expression.

### siRNA mediated silencing of human S1P_1_, S1P_2_ and S1P_3_

LN18 cells were transfected using the Lipofectamine^®^ 2000 reagent protocol (Invitrogen). Gene specific siRNA and control siRNA (both OriGene Technologies, USA) were used at a final concentration of 5 pmol. To optimize the knockdown effectivity the whole transfection procedure was repeated 24 h after the first application of the siRNA. RNA knockdown was tested 48 h post-transfection using quantitative real-time PCR.

### Cell viability analysis

LN18 cells were seeded at a density of 10 000 cells/well onto 96-well multiplates. 24 hours later, the medium was changed and cells were incubated for different time points with fresh medium containing S1P or the respective S1P receptor inhibitor. Afterwards, medium was removed and replaced by fresh medium containing 10% resazurine (PromoCell, Heidelberg, Germany), and cells were further incubated for 2 h at 37°C. Fluorescence signals were recorded using a multiplate reader (Tecan Infinite M200, Crailsheim, Germany, excitation, 530 nm; emission, 590 nm) and data were calculated as percentage of cell viability of solvent (MeOH for S1P, DMSO for inhibitors) treated cells.

### Scratch wound healing assay

For the wound healing assay, 150 000 cells/well were seeded onto 24-well multiplates. When cells reached confluency, a defined wound scratch was set into the middle of the cell layer using a yellow pipet tip. Detached cells were removed by washing with PBS followed by application of 5 mM hydroxy urea as proliferation inhibitor. Using the PALM Robo Software of the AxioVision HXP120C microscope (Carl Zeiss Microscopy, Jena, Germany) the wound was imaged and the exact position of the image was saved to analyze the same region after the respective incubation time. After the pre-incubation with hydroxy urea for 1 h, cells were treated with the different inhibitors and/or S1P for 16 h followed by the analysis of the wound width (Software AxioVision SE64 Rel. 4.9, Carl Zeiss Microscopy).

### Boyden chamber assay

Using a Boyden chamber (Neuro Probe Standard Chemotaxis Chamber, Neuro Probe Inc., Gaithersburg, USA) we performed a transwell migration assay. Briefly, cells were cultured for 24 h in serum-free media followed by trypsinization and seeding of 5 000 cells/50 μl FCS-free media into the upper well of the Boyden chamber. A polycarbonate membrane with a pore size of 8 μm (Whatman GmbH, Dassel, Germany) was located between the upper and the bottom chamber. Cells were treated for 3 h with the respective inhibitor and/or S1P. Afterwards, migrated cells were fixed on the lower side of the membrane with 4% paraformaldehyde, stained with crystal violet solution for 30 min and counted using the cell counting tool of ImageJ (National Institute of Health, Maryland, USA).

### Statistical analysis

Statistical analyses were performed with GraphPad Prism 5.0. (GraphPad Software, Inc., California, USA). Data of *in vitro* analyses represent 3 or 4 independent experiments (indicated in the figure legends and shown as mean ± SD). Box plots of data of patients’ samples are shown as the median and the 5th and 95th percentiles. Pairwise comparisons were performed using Mann–Whitney *U* test. More than 2 groups were compared by OneWay ANOVA and Dunnett's multiple comparison test. The duration of a patient's overall survival (OS) was defined as the time from the first tumor detection until death. Information on vital status and date of death were obtained from official population registry. Based on the gene expression, GBM specimens were divided into the lower half versus the upper half of gene expression level as determined by real-time PCR (< Median vs. > = Median expression). These were used for calculation of Hazard Ratios (< Median vs. > = Median expression) and creation of Kaplan–Meier graphs which were compared by log-rank (Mantel-Cox) test. Specimens with expression rates lower than the detection limit of quantitative real-time PCR were excluded from data analysis. Statistical significances were defined as *p < 0.05, **p < 0.01, and ***p < 0.001.

## SUPPLEMENTARY MATERIALS FIGURES


