# Supplementary material for: Expression of S1P metabolizing enzymes and receptors correlate with survival time and regulate cell migration in glioblastoma multiforme
Source: Oncotarget. 2016 Feb 13;7(11):13031–46. doi: 10.18632/oncotarget.7366 (PMC4914339; doi:10.18632/oncotarget.7366)
Supplement: Supplementary file 1 [file oncotarget-07-13031-s001.pdf]

Expression of S1P metabolizing enzymes and receptors correlate with survival time and regulate cell migration in glioblastoma multiforme

Supplementary Materials

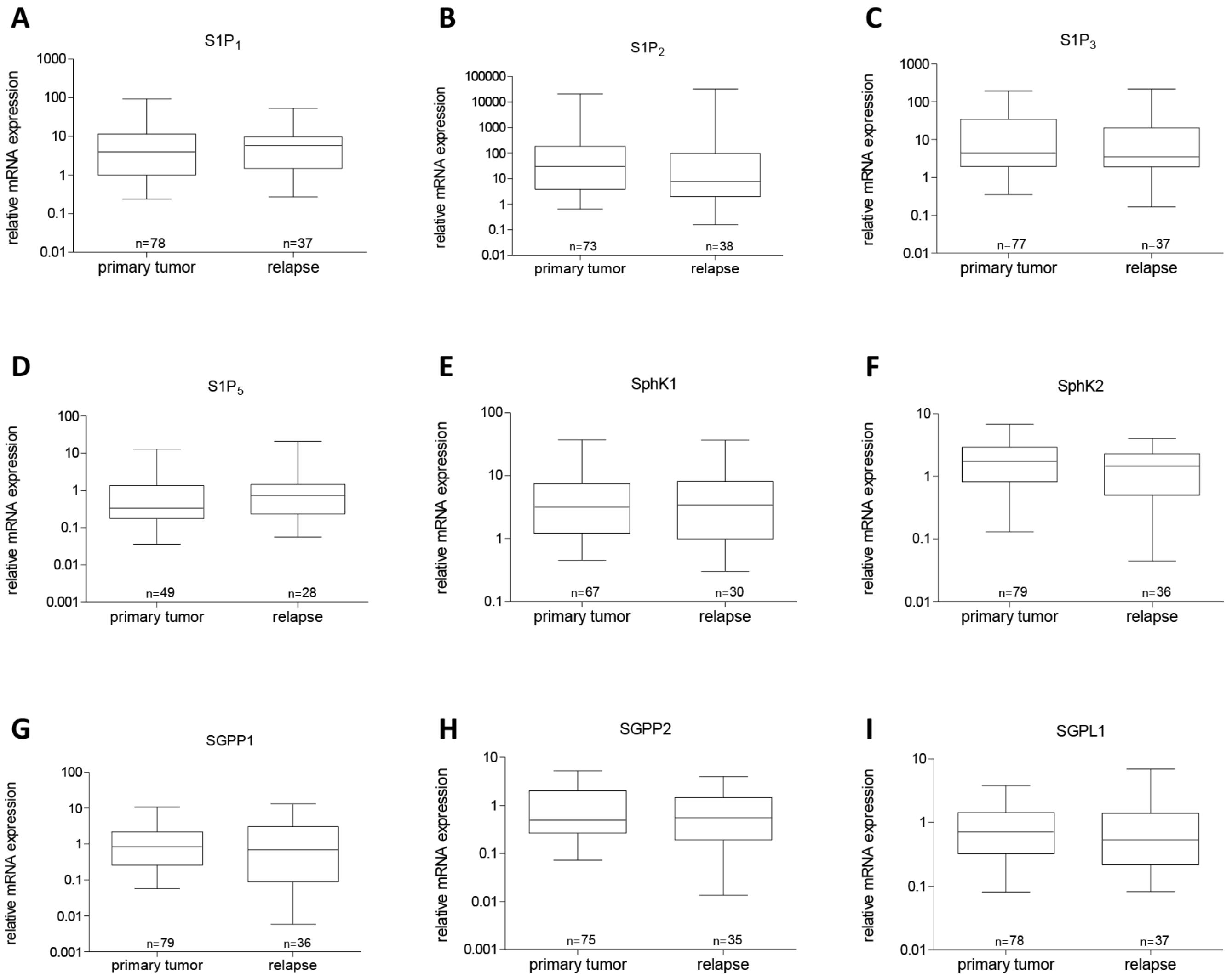

Supplementary Figure S1: mRNA expression of S1P receptors and S1P metabolizing enzymes in primary glioblastoma in comparison to relapses. (**A**) $S1P_1$ mRNA expression, (**B**) $S1P_2$ mRNA expression, (**C**) $S1P_3$ mRNA expression, (**D**) $S1P_5$ mRNA expression, (**E**) SphK1 mRNA expression, (**F**) SphK2 mRNA expression, (**G**) SGPP1 mRNA expression, (**H**) SGPP2 mRNA expression, (**I**) SGPL1 mRNA expression. mRNA expression levels in glioblastoma patient´s samples were analyzed by quantitative RT-PCR with normalization to 18S rRNA. Data are shown as box plots representing the median as horizontal bars as well as the 5th and 95th percentile.

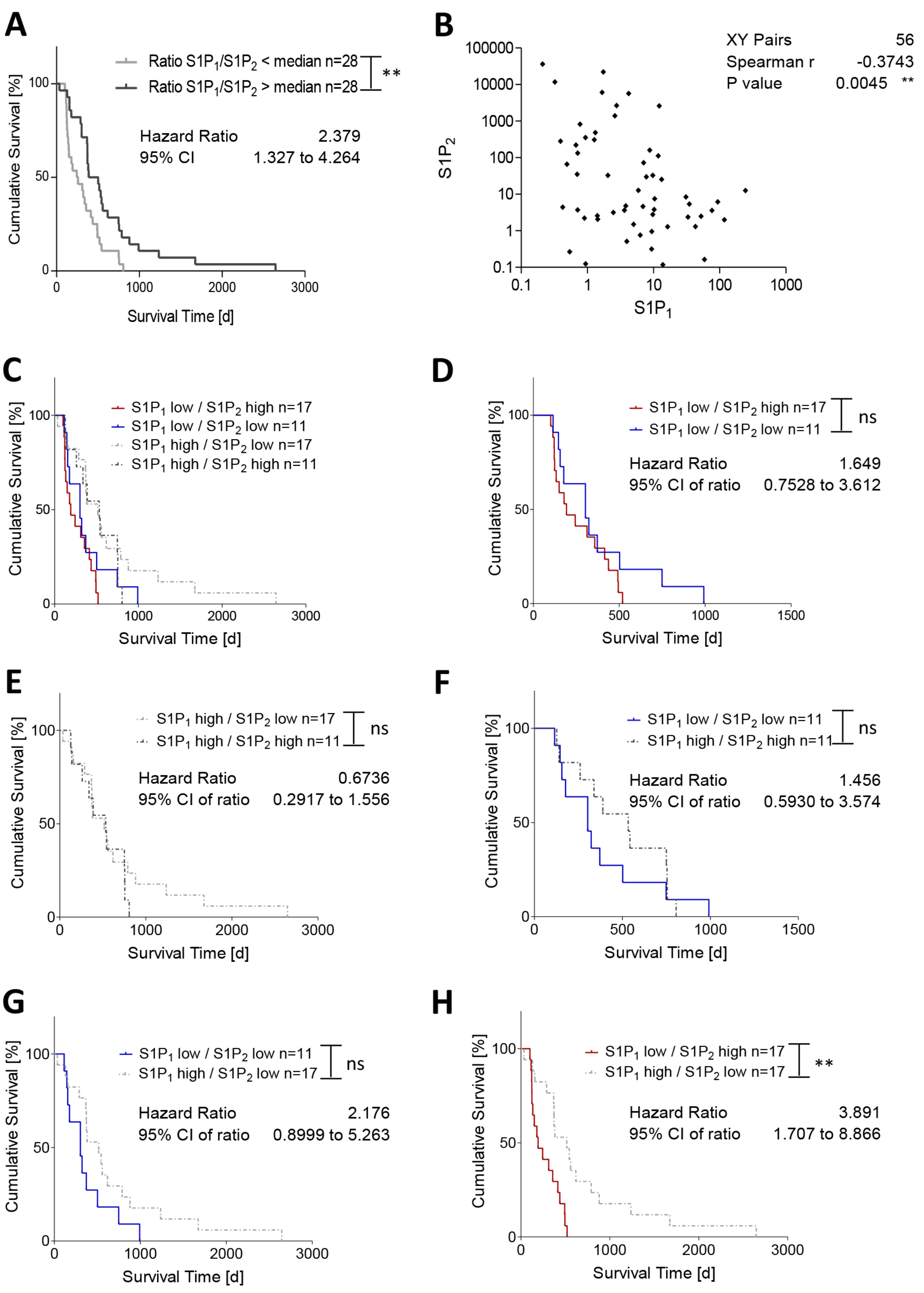

Supplementary Figure S2: Combined evaluation of the impact of S1P$_1$ and S1P$_2$ on survival time of patients with GBM. (**A**) Kaplan-Meier survival curves for patients with GBM based on their S1P$_1$/S1P$_2$ ratio. Patients were divided into two subgroups depending on the respective median S1P$_1$/S1P$_2$ ratio of gene expression as determined by quantitative RT-PCR. Log-rank (Mantel-Cox) test, $**p < 0.005$. (**B**) Spearman's nonparametric correlation analysis of S1P$_1$ and S1P$_2$ gene expression in 56 GBM patient samples ($r = -0.37$, $p = 0.0045$). (**C**) Kaplan-Meier survival curves for GBM patients with dividing patients into four subgroups depending on both S1P$_1$ and S1P$_2$ mRNA expression as determined by quantitative RT-PCR. Subdivision is based on the median gene expression with values < Median (low) and > Median (high). (**D**–**H**) Separate presentation of the Kaplan-Meier survival curves of (C). Log-rank (Mantel-Cox) test, $**p < 0.005$ (ns = not signficant).

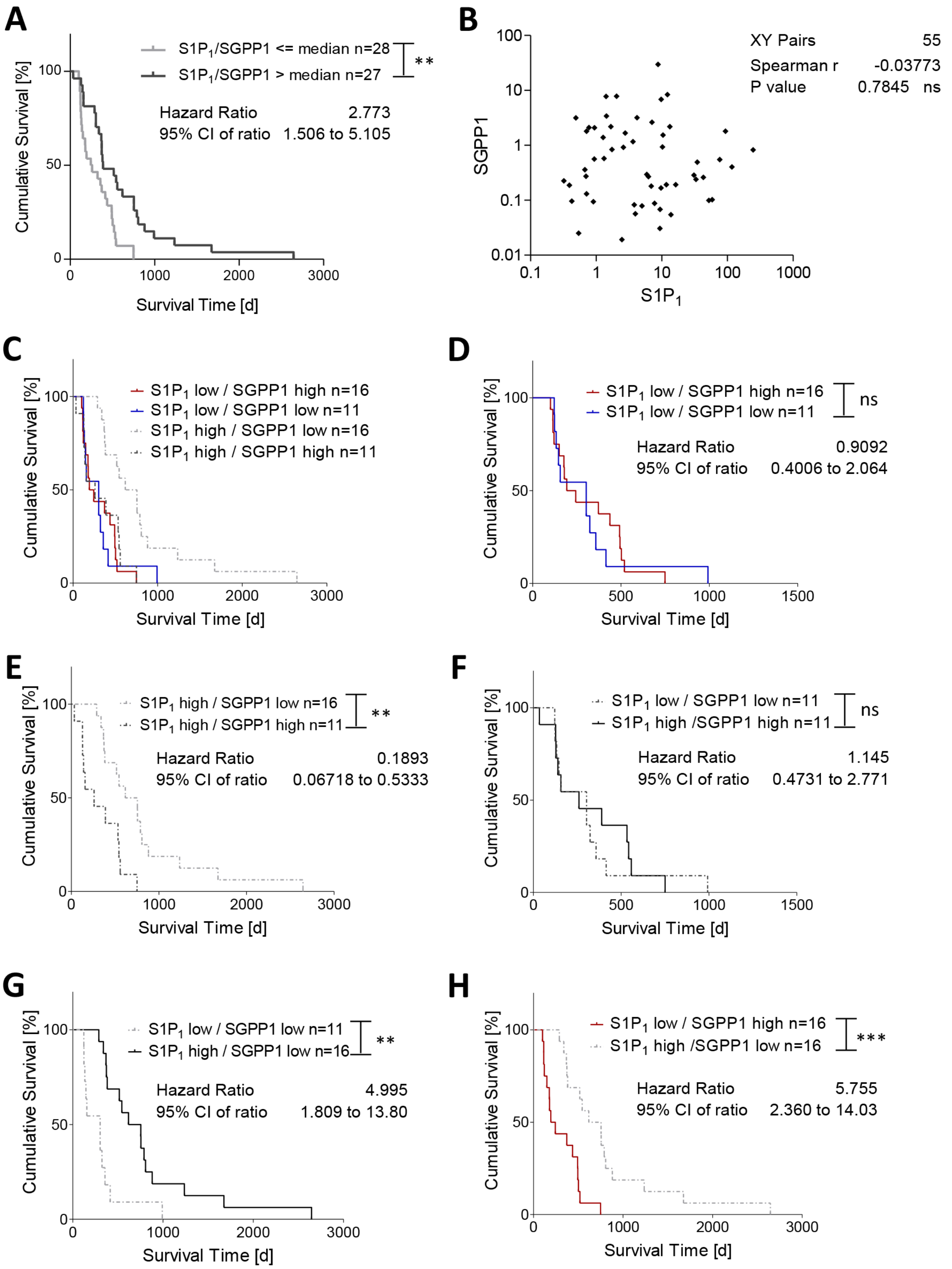

Supplementary Figure S3: Combined evaluation of the impact of $S1P_1$ and SGPP1 on survival time of patients with GBM. (**A**) Kaplan-Meier survival curves for patients with GBM based on their S1P1/SGPP1 ratio. Patients were divided into two subgroups depending on the respective median $S1P_1$/SGPP1 ratio of gene expression as determined by quantitative RT-PCR. Log-rank (Mantel-Cox) test, $^{**}p < 0.005$. (**B**) Spearman's nonparametric correlation analysis of $S1P_1$ and SGPP1 gene expression in 56 GBM patient samples ($r = -0.038$, $p = 0.785$). (**C**) Kaplan-Meier survival curves for GBM patients with dividing patients into four subgroups depending on both $S1P_1$ and SGPP1 mRNA expression as determined by quantitative RT-PCR. Subdivision is based on the median gene expression with values < Median (low) and > Median (high). (**D**–**H**) Separate presentation of the Kaplan-Meier survival curves of (C). Log-rank (Mantel-Cox) test, $^{**}p < 0.005$, $^{***}p < 0.001$ (ns = not significant).

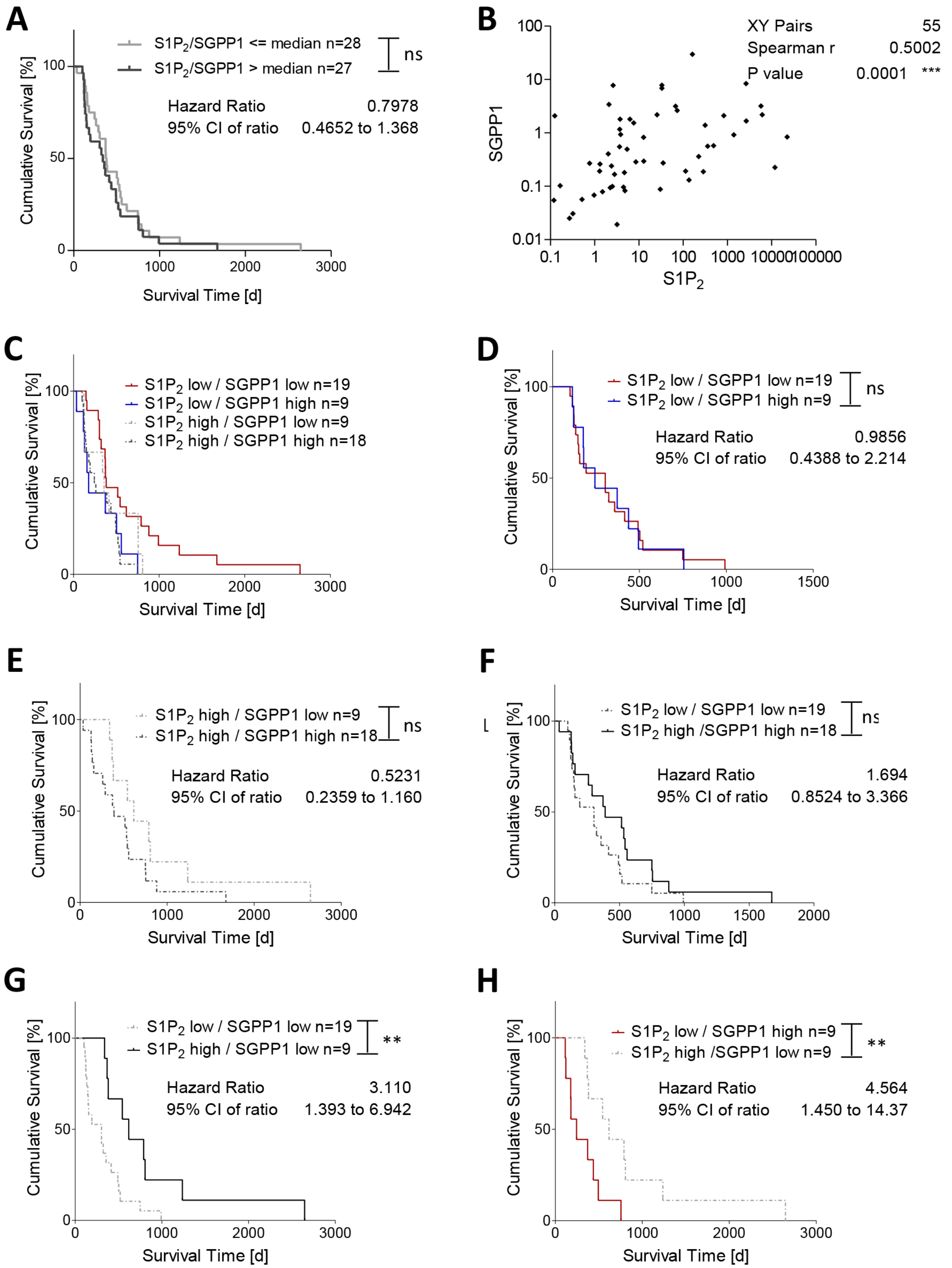

Supplementary Figure S4: Combined evaluation of the impact of S1P$_2$ and SGPP1 on survival time of patients with GBM. (**A**) Kaplan-Meier survival curves for patients with GBM based on their S1P$_2$/SGPP1 ratio. Patients were divided into two subgroups depending on the respective median S1P$_2$/SGPP1 ratio of gene expression as determined by quantitative RT-PCR. Log-rank (Mantel-Cox) test. (**B**) Spearman's nonparametric correlation analysis of S1P$_2$ and SGPP1 gene expression in 56 GBM patient samples ($r = 0.05$, $p = 0.0001$). (**C**) Kaplan-Meier survival curves for GBM patients with dividing patients into four subgroups depending on both S1P$_2$ and SGPP1 mRNA expression as determined by quantitative RT-PCR. Subdivision is based on the median gene expression with values < Median (low) and > Median (high). (**D**–**H**) Separate presentation of the Kaplan-Meier survival curves of (C). Log-rank (Mantel-Cox) test, $^{**}p < 0.005$ (ns = not significant).

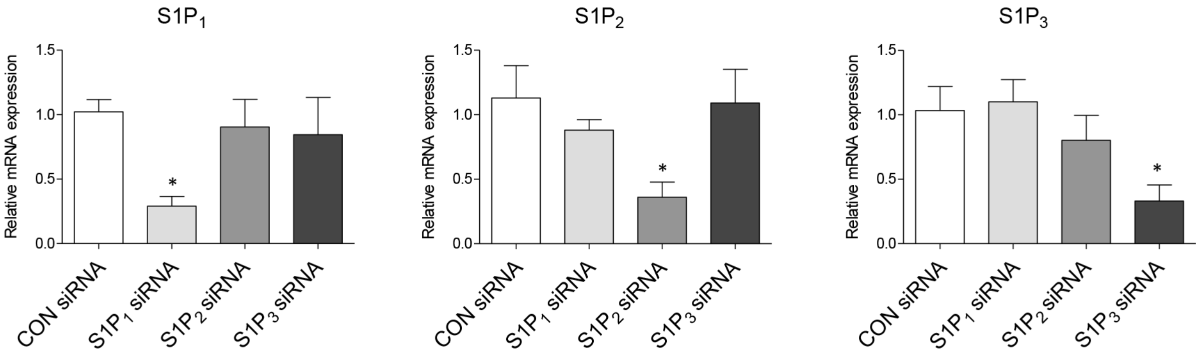

Supplementary Figure S5: mRNA expression analysis of the three investigated S1P receptors ($S1P_{1–3}$) after specific siRNA-mediated down-regulation of either $S1P_1$, $S1P_2$ or $S1P_3$. Relative mRNA expression of S1P receptors in the human LN18 GBM cell line was determined by quantitative RT-PCR with normalization to the mean of 18S rRNA, GAPDH and TBP expression. Control cells (CON siRNA) were transfected with a non-targeting siRNA, mean values and SD ($n = 3$), One-way analysis of variance with Dunnett´s multiple comparison test, *$p < 0.05$ vs. CON siRNA.
